# Dermoscopy of Circumscribed Acral Hypokeratosis

**DOI:** 10.5826/dpc.1101a87

**Published:** 2020-12-07

**Authors:** John J. Dávila-Rodríguez, Liliana García, Diana Posso, Giuseppe Argenziano

**Affiliations:** 1 Department of Dermatologic Surgery, Dermatologic Institute of Jalisco Dr. José Barba Rubio, Guadalajara, Mexico; 2 Department of Dermatology, Carlos Andrade Marín Hospital, Quito, Ecuador; 3 Department of Pathology, Carlos Andrade Marín Hospital, Quito, Ecuador; 4 Dermatology Unit, University of Campania, Luigi Vanvitelli, Naples, Italy

**Keywords:** dermoscopy, circumscribed acral hypokeratosis, soles

## Introduction

Circumscribed acral hypokeratosis (CAH) was recently described as a rare benign condition affecting the palms and soles of middle-aged patients that represents a possible localized abnormal keratinization [1].

## Case Presentation

**Figure 1:**
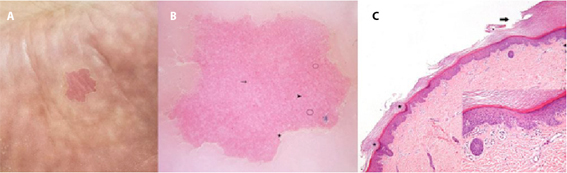
(A) Clinical presentation of circumscribed acral hypokeratosis. (B) Dermoscopic description: Stair-step desquamation at the periphery (star), dotted vessels (arrowhead), white dots (arrow) and rosettes (circles) in a linear distribution along the ridges. (C) Stair-like thinning of the horny layer toward the center (arrow), protrusion of acrosyringeal corneocytes (stars) (H&E, × 10). The inset shows hypogranulosis under the hypokeratotic area and dilated congestive capillaries in the papillary and reticular dermis (H&E, × 40).

A 68-year-old woman presented with an asymptomatic plantar lesion that had evolved over 10 years. On physical examination, a 1.5-cm erythematous patch on the plantar medial arch was seen with evident irregular and scaling edges (Figure 1A). Dermoscopy showed a stair-step desquamation at the periphery of the lesion and dotted vessels with a diffuse erythematous background scattered with white dots in linear arrangement on the ridges. In addition, several rosettes were observed on the ridges (Figure 1B). Histopathology showed stairlike thinning of the horny layer towards the center, protrusion of acrosyringeal corneocytes, and hypogranulosis under the hypokeratotic area. Furthermore, dilated congestive capillaries were present in the papillary and reticular dermis (Figure 1C). This lesion was successfully treated with surgical excision.

**Figure 2:**
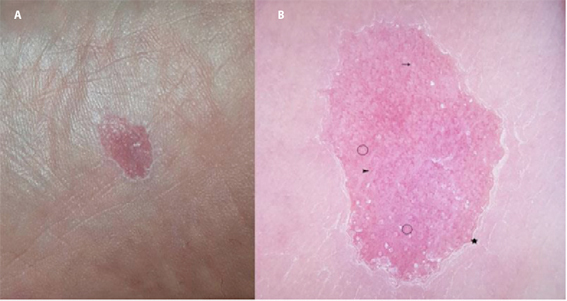
(A) Clinical and (B) dermoscopic description: stair-step desquamation (star), dotted vessels (arrowhead), white dots (arrow), and rosettes (circles).

The second patient, a man in his 70s, presented with a 15-year history of an asymptomatic erythematous, scaly, well-demarcated plaque on the plantar medial arch (Figure 2A). Dermoscopy showed a stair-step configuration of the border, a parallel white crista dotted pattern with some rosettes, and a homogeneous erythematous area with dotted vessels (Figure 2B). Histopathologic examination showed similar features as in the case above, and the patient received the same treatment.

Dermoscopic findings in our 2 cases are similar to those of Topin et al [1]. We noted that the stair-step dermoscopic configuration at the periphery of the lesion correlates to stepped thinning of the stratum corneum towards the center. Moreover, white dots correspond to acrosyringia that were accentuated as a result of the reduction in the horny layer. We used the term “parallel white crista dotted pattern” to describe the linear disposition of these structures on the ridges. This configuration is explained by the eccrine duct anatomy, which opens to the skin’s surface through the crista superficialis.

We suggest that the diffuse erythema may be the result of vessels becoming visible through the transparent and thin stratum corneum but also to the presence of dilated vessels at the papillary and upper reticular dermis. The congestive vessels correspond to dotted vessel pattern dermoscopically. We observed that this pattern tended to follow a linear configuration mainly on the furrows but occasionally on the ridges, too.

The presence of rosettes in CAH may be caused by the presence of concentric horny material in the acrosyringium. It could be that rosettes are more common in areas with marked protrusion of acrosyringeal corneocytes. It is currently known that rosettes are seen exclusively with polarized dermoscopy that is probably attributable to an optical effect. These structures are not specific and can be observed in a range of conditions from tumoral to inflammatory skin lesions. In Table 1 the main dermoscopic differential diagnoses of CAH are listed.

## Conclusions

Dermoscopy can be useful in the diagnosis of CAH and can help in differentiating this disease from other dermatoses affecting the palmoplantar areas. Rosettes can be present and may correlate with a marked protrusion of acrosyringeal corneocytes. We propose the term “parallel white crista dotted pattern” in order to describe the linear arrangement of white dots and rosettes on the ridges.

## Figures and Tables

**Table 1:** Differential Diagnoses of CAH [1,2].

Feature	CAH	Bowen Disease	Mibelli Porokeratosis
**Scale**	Peripheral stair-step	Scaly surface	Peripheral double rim
**Central Part**	Erythematous and homogeneous	Erythematous with yellow crusts, focal hemorrhage, focal/multifocal hypopigmentation	Hypopigmented
**Vessel Pattern**	Dotted in linear arrangement mainly on the furrows	Glomerular, arranged in clusters, linear, irregular or dotted	Dotted
**Dots**	White in linear arrangement on the ridges	Gray, brown in patchy or linear arrangement	Red-brown
**Rosettes**	Yes	Yes	No

CAH = circumscribed acral hypokeratosis
